# Use of a physiologically-based pharmacokinetic model to simulate artemether dose adjustment for overcoming the drug-drug interaction with efavirenz

**DOI:** 10.1186/2193-9616-1-4

**Published:** 2013-03-01

**Authors:** Marco Siccardi, Adeniyi Olagunju, Kay Seden, Farid Ebrahimjee, Steve Rannard, David Back, Andrew Owen

**Affiliations:** 1 Department of Molecular and Clinical Pharmacology, Institute of Translational Medicine, University of Liverpool, Liverpool, UK; 2 Faculty of Pharmacy, Obafemi Awolowo University, Ile-Ife, Nigeria; 3 Department of Chemistry, University of Liverpool, Liverpool, UK

**Keywords:** Artemether, IVIVE, Efavirenz, Drug interaction, Pharmacokinetics, Simulation

## Abstract

**Purpose:**

To treat malaria, HIV-infected patients normally receive artemether (80 mg twice daily) concurrently with antiretroviral therapy and drug-drug interactions can potentially occur. Artemether is a substrate of CYP3A4 and CYP2B6, antiretrovirals such as efavirenz induce these enzymes and have the potential to reduce artemether pharmacokinetic exposure. The aim of this study was to develop an *in vitro in vivo* extrapolation (IVIVE) approach to model the interaction between efavirenz and artemether. Artemether dose adjustments were then simulated in order to predict optimal dosing in co-infected patients and inform future interaction study design.

**Methods:**

*In vitro* data describing the chemical properties, absorption, distribution, metabolism and elimination of efavirenz and artemether were obtained from published literature and included in a physiologically based pharmacokinetic model (PBPK) to predict drug disposition simulating virtual clinical trials. Administration of efavirenz and artemether, alone or in combination, were simulated to mirror previous clinical studies and facilitate validation of the model and realistic interpretation of the simulation. Efavirenz (600 mg once daily) was administered to 50 virtual subjects for 14 days. This was followed by concomitant administration of artemether (80 mg eight hourly) for the first two doses and 80 mg (twice daily) for another two days.

**Results:**

Simulated pharmacokinetics and the drug-drug interaction were in concordance with available clinical data. Efavirenz induced first pass metabolism and hepatic clearance, reducing artemether C_max_ by 60% and AUC by 80%. Dose increases of artemether, to correct for the interaction, were simulated and a dose of 240 mg was predicted to be sufficient to overcome the interaction and allow therapeutic plasma concentrations of artemether.

**Conclusions:**

The model presented here provides a rational platform to inform the design for a clinical drug interaction study that may save time and resource while the optimal dose is determined empirically. Wider application of IVIVE could help researchers gain a better understanding of the molecular mechanisms underpinning variability in drug disposition.

## Background

The geographical overlap in the prevalence, morbidity and mortality of malaria and HIV constitutes a major public health burden in low- and middle-income countries. In 2010 alone, more than 34 million people were living with HIV worldwide (68% in Sub-Saharan Africa) and approximately 1.8 million died of AIDS-related illnesses (UNAIDS 2010). Malaria caused an estimated 219 million acute illnesses and 660,000 deaths in the same year (WHO 2012). Approximately 80% of these cases and 90% of deaths occurred in Sub-Saharan Africa, disproportionately affecting children less than 5 years old and pregnant women (WHO 2012). Jointly accounting for about 2.5 million deaths in 2010 alone, there is little doubt over the public health consequences of these diseases.

Limited access to highly active antiretroviral therapy (HAART) in low and middle income countries constitutes a major barrier that substantially diminishes the number of therapeutic options available. Therefore, protection of currently available first-line antiretroviral and antimalarial drugs against resistance is of paramount importance (Vella et al. 2012). HAART is based on the combination of different classes of drugs and in resource-limited countries (as elsewhere), regimens including the non-nucleoside reverse transcriptase inhibitors (NNRTIs), nevirapine and efavirenz, are indicated as first-line therapies.

Artemether/lumefantrine, the first-line artemisinin-based combination therapy (ACT) for treatment of malaria, is currently administered concomitantly with HAART in co-infected patients in several African countries. A six-dose regimen administered over three days has excellent efficacy against *plasmodium falciparum* malaria. Co-infection with malaria and HIV has been shown to impact negatively on the course of both infections (WHO 2005). Gonzalez *et al*. recently provided a comprehensive review of the epidemiological, clinical, immunological and therapeutic interactions between malaria and HIV (Gonzalez et al. 2012). Unfortunately, available treatment options in high burden countries are limited and development of effective treatment strategies that protect available first-line drugs is paramount as second line drugs are more costly and frequently unavailable.

Artemether is a substrate for cytochrome P450 3A4 (CYP3A4) and CYP2B6, and is quickly absorbed in the first two to three hours after oral administration. Bioavailability is low due to intestinal and first-pass hepatic metabolism (Byakika-Kibwika et al. 2012). Antiretrovirals induce or inhibit hepatic CYPs and therefore have the potential to cause several drug drug interactions. More specifically, boosted PI can cause significant drug interactions due to the potent inhibition of CYP3A4 by ritonavir (RTV) and efavirenz and nevirapine can induce the expression of several CYPs. Drug-drug interactions have been described for several class of drugs such as, immunosuppressants, statins, antipsychotics, antifungals and antibacterials. (Marzolini et al. 2010). EFV is thought to reduce bioavailability and increase hepatic clearance of artemether, thereby reducing plasma exposure. Recently, Byakika-Kibwika *et al.* reported 59% and 79% reductions in artemether C_max_ and plasma AUC, respectively, when co-administered with efavirenz in HIV-infected adults (Byakika-Kibwika et al. 2012). Similarly, Huang *et al*. reported a 51% decrease in artemether AUC when co-administered with efavirenz in healthy volunteers (Huang et al. 2012). Since efavirenz-containing regimens are preferred for patients initiating therapy, dose optimisation strategies to mitigate the interaction are worthy of investigation (Best and Goicoechea 2008).

*In vitro in vivo* extrapolation (IVIVE) is a bottom up technique which aims to simulate pharmacokinetics using *in vitro* data, such as the physicochemical characteristics and intrinsic clearance (CL_int_) through physiologically-based pharmacokinetic (PBPK) models, which mathematically describe absorption, distribution, metabolism and elimination (ADME). Therapeutic agents can be administered via different routes and absorption can be simulated taking into account the dynamic interplay between dissolution, passive permeability and affinity/activity of metabolic enzymes and transporters. For instance, oral bioavailability (F_oral_) can be influenced by tablet dissolution and solubility, impacting the fraction of dose available for absorption (F_a_), whereas intestinal (F_g_ = fraction of dose available following intestinal metabolism) and first-pass hepatic metabolism (F_h_ = fraction of dose available following hepatic metabolism) can reduce the amount of drug reaching the systemic circulation (Kimura et al. 2000). F_oral_ can be evaluated using compartmental absorption and transit (CAT) models estimating the fraction of dose absorbed and the rate of drug absorption based on transit models. Volume of distribution is simulated by evaluating tissue volumes and the diffusion of drugs into tissues, which is influenced by physicochemical properties defining the plasma to tissue ratio (P_t:p_) as described by Poulin and Theil (Poulin and Theil 2002). Metabolism of drugs is the result of the activity of several metabolic enzymes in different tissues. I*n vitro* intrinsic clearance data can be used to simulate hepatic clearance considering scaling factors such as CYP isoform abundance in microsomal protein, microsomal protein per gram of liver, liver weight, blood flow and protein binding. Inter-patient variability is observed in all the above mentioned processes and PBPK models allow the introduction of anatomical and physiological characteristics and their covariance to build a virtual population of patients. Consequently, PBPK models give insight into mechanisms regulating the pharmacokinetics, can be used to predict potential variability in populations and to simulate the pharmacokinetic consequences of different dose strategies.

The aim of this study was to develop an IVIVE model for efavirenz and artemether pharmacokinetics and simulate a clinical drug interaction trial. The IVIVE model was validated by comparison with existing clinical data and then used to simulate appropriate artemether dose adjustments able to achieve therapeutic artemether exposure when co-administered with efavirenz.

## Methods

### *In vitro-in vivo* extrapolation: system parameters

**Figure 1:**
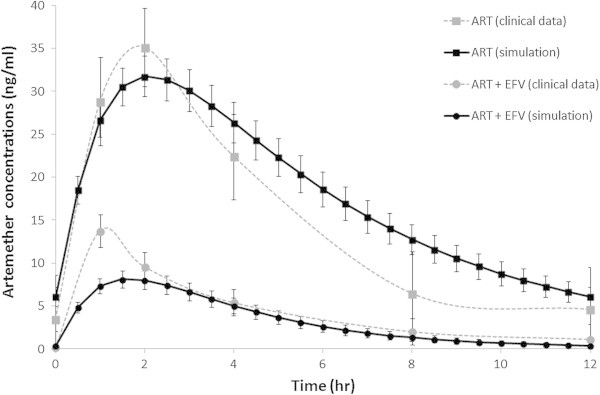
**Schematic representation of the physiologically based pharmacokinetic (PBPK) model structure including the most relevant organs and the blood circulation.**

Virtual patients were generated using a population physiology model (*physB*), which provides a statistical description of the physiological and anatomical characteristics in the human population, focusing on parameters that are essential in the PBPK approach (Bosgra et al. 2012). As summarised in Table 1, organ weights were allometrically scaled to an individual’s height or BSA (for skin and blood) or age plus gender (brain) or height and body weight (adipose tissues) and random variation was added to all parameters. Cardiac output has been defined as QC = QCC × (body weight) ^0.75^ and regional blood flows were simulated as previously described (International Life Sciences Institute and Risk Science Institute 1994). A schematic of a PBPK model is shown in Figure 1. The differential equations used in these models to simulate drug distribution in tissues have been described previously (Jones et al. 2006) and can be represented as:1

where Q = blood flow, C = concentration, V = volume, T = tissues, ab = arterial, Kp = tissue to plasma partition coefficient, and B:P = blood to plasma ratio.

**Table 1:** **Main model parameters and equations used to simulate organ weight and regional cardiac output**

Model parameter	Equation	Standard deviations
BSA	0.007184 x BW^0.425^x H^0.725^	
W_blood_	3.33 x BSA-0.81 (male)	0.10
	2.66 x BSA-0.46 (female)	
W_lungs_	10^(−2.092 + 2.1 x logH)^	0.195
W_heart_	10^(−2.502 + 2.13 x logH)^	0.069
W_bones_	10^(0.0689 + 2.67 x logH)^	0.116 (m); 0.083 (f)
W_kidney_	10^(−2.306 + 1.93 x logH)^	0.140
W_stomach_	10^(−3.266 + 2.45 x logH)^	0.0965 (m); 0.0425 (f)
W_intestine_	10^(−1.351 + 2.47 x logH)^	0.049
W_spleen_	10^(−3.123 + 2.16 x logH)^	0.156
W_pancreas_	10^(−3.431 + 2.43 x logH)^	0.245 (m); 0.087 (f)
W_liver_	10^(−0.6786 + 1.98 x logH)^	0.028 (m); 0.048 (f)
W_remaining_	10^(−0.072 + 1.95 x logH)^	0.049
W_brain_	B x {[(3.68 – 2.68 x e^(−age/0.89)^] x e^(−age/629)^]}	0.084
W_skin_	e^1.64xBSA-1.93^	0.049
W_adipose_	(1.20 x BMI) – (0.7 x age) – (3.6 x gender) + 1.4	
W_muscle_	remains	
CO	QCC × (BW) ^0.75^	
QC_adipose_	CO x 0.052	
QC_bones_	CO x 0.042	
QC_brain_	CO x 0.114	
QC_kidney_	CO x 0.175	
QC (total)_liver_	CO x 0.227	
QC (portal)_liver_	CO x 0.181	
QC_muscle_	CO x 0.191	
QC_skin_	CO x 0.058	

H = height; B = brain weight at birth (405 g for males and 373 g females); BW = body weight; QC = blood flow; CO = cardiac output.

Expression of cytochrome P450 in hepatic and intestinal tissue was taken from previous reports (Harbourt et al. 2012 ; Houston 1994 ; Ohtsuki et al. 2012). Oral bioavailability is influenced by several processes. Experimental data such as apparent permeability in Caco-2 cell monolayer or polar surface area and number of hydrogen bond donor atoms have been used to derive effective permeability (P_eff_) and subsequently the constant of absorption (ka) (Gertz et al. 2010). Tablet dissolution and poor solubility can impact the fraction of dose available for absorption (F_a_), and intestinal (F_h_) and first-pass hepatic metabolism (F_g_) can reduce the amount of drug reaching the systemic circulation. Tablet dissolution for artemether was taken into account using a first-order dissolution constant as previously described (Awofisayo et al. 2012). F_g_ and F_h_ were evaluated using the following equations (Kimura et al. 2000):23

Where Q_h,portal_, Q_g,_ Fu, CL_int,h_, CL_int,g_ represent portal blood flow to liver, blood flow to intestine, fraction unbound in tissue, hepatic intrinsic clearance and intestinal intrinsic clearance, respectively.

Oral absorption was simulated using a compartmental absorption and transit model and considering a stomach transit time of 0.5 hours and a small intestine transit time of 3.3 hours as previously described (Yu and Amidon 1999). The volume of distribution was simulated using the equation developed by Poulin and Theil, calculating the plasma to tissue ratio for each organ and considering organ volumes originated from the population physiology model () (Poulin and Theil *physB*^2002^). Hepatic clearance (CL_h_) and induction of enzyme expression in liver (E) were determined using the following equations (Obach 1999):45

Where Q_h_, F_u_, CL_int,h_, E_MAX_, EC_50_ and I_h_ represent blood flow to the liver, fraction unbound in blood, hepatic intrinsic clearance, maximum induction, concentration causing 50% of maximum induction and inducer concentrations in the liver tissue, respectively. Induction of metabolism enzymes was corrected to achieve 100% induction in 14 days.

### *In vitro-in vivo* extrapolation: drug parameters

Efavirenz and artemether pharmacokinetics were simulated using an open source PBPK model developed using Berkeley Madonna (version 8.3.18, University of California, CA, USA). *In vitro* data describing efavirenz and artemether physiochemical and metabolic characteristics and induction potential are summarized in Table 2.

**Table 2:** **Efavirenz and artemether physiochemical and metabolic characteristics**

Input parameter	Efavirenz	Arthemether
logP	4.6 (DRUGBANK - Efavirenz)	3.4 (DRUGBANK - Artemether)
PSA		46.15 (DRUGBANK - Artemether)
Caco-2 Papp	2.5 (10^-6^ cm/s) (Siccardi et al. 2012)	
fu	0.01 (Almond et al. 2005)	0.05 (DRUGBANK - Artemether)
**Metabolism (μl/min/pmol)**		
rCYP2B6 CL_int_	0.55 (Ward et al. 2003)	9.31 (Honda et al. 2011)
rCYP1A2 CL_int_	0.07 (Ward et al. 2003)	
rCYP2A6 CL_int_	0.08 (Ward et al. 2003)	
rCYP3A4 CL_int_	0.007 (Ward et al. 2003)	1.47 (Honda et al. 2011)
rCYP3A5 CL_int_	0.03 (Ward et al. 2003)	
**CYP induction**		
CYP2B6 E_max_	5.7 (Rekic et al. 2011)	
CYP2B6 EC_50_	0.8 (Rekic et al. 2011)	
CYP3A4 E_max_	6.5 (Rekic et al. 2011)	
CYP3A4 EC_50_	3.9 (Rekic et al. 2011)	

MW, molecular weight; logP, Logarithm of the Octanol-water partition coefficient; pKa, Acid dissociation constant; fu, fraction unbound in plasma; Papp, apparent permeability; Clint, intrinsic clearance; Indmax, Maximum induction; IndC50, inducer concentration that supports half maximal induction (μM).

For artemether, tablet dissolution and the poor solubility described in a previous publication were included in the model (Awofisayo et al. 2012). Data describing the metabolism of efavirenz and artemether by different recombinant enzyme isoforms were obtained from the literature (Belanger et al. 2009 ; Ogburn et al. 2010 ; Ward et al. 2003). As described in Table 2, *in vitro* intrinsic clearance (CL_int_) for the two compounds were included in the model: efavirenz is hydroxylated to 7-hydroxy efavirenz by CYP2A6, to 8-hydroxy efavirenz by CYP2B6, CYP2A6, CYP1A2, CYP3A4 and CYP3A5, and glucuronidated by UGT2B7 while artemether is metabolised by CYP2B6 and CYP3A4. After correction with fraction unbound in microsomes ( reaction (Poulin and Haddad measured experimentally or simulated as previously described for non-specific binding in the *in vitro*^2011^)) these data were scaled up to hepatic or intestinal intrinsic clearance considering the amount of microsomal protein per gram of liver and liver weight or total content of CYP3A4 in the intestine (Crewe et al. 2011 ; Proctor et al. 2004).

### Virtual clinical study design

Administration of efavirenz and artemether, alone or in combination, were simulated to mirror previous clinical studies and facilitate validation of the model and realistic interpretation of the simulation (Byakika-Kibwika et al. 2012 ; Huang et al. 2012). Efavirenz (600 mg once daily) was administered to 50 virtual subjects (20–50 years old, 0.5 proportion females) for 14 days. This was followed by concomitant administration of artemether (80 mg eight hourly) for the first two doses and 80 mg (twice daily) for another two days. The simulated pharmacokinetics of efavirenz and artemether were compared to previous experimental findings. Subsequently, artemether dose adjustment was simulated to establish doses attaining artemether exposure equivalent to that in the absence of efavirenz.

## Results and discussion

**Figure 2:**
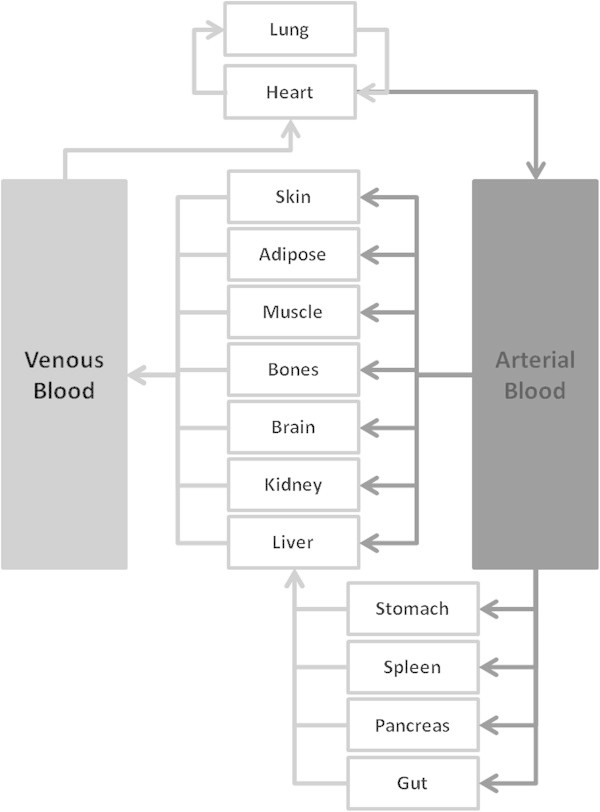
**Simulated artemether concentration-time profile for a dose of 80 mg twice daily with and without efavirenz.** The full black line represents the mean (± SE) simulated concentrations and grey lines represent data observed in a clinical study (Byakika-Kibwika et al. 2012).

Simulated pharmacokinetics of artemether and efavirenz were in good accordance with previously described clinical data, as represented in Table 3 and Figure 2 (Byakika-Kibwika et al. 2012 ; Huang et al. 2012). In a virtual cohort of 50 patients treated with 80 mg of artemether twice daily for three days the simulated median (range) AUC was equal to 166 (55–678) vs 119 (26–917) ng/ml•h (reference value), C_max_ 30 (11–73) vs 29 (10–247) ng/ml. Simulation of artemether drug disposition was characterised by low bioavalability (F_oral_ = 0.11), with around 14% of the dose not absorbed (F_a_ = 0.86), high intestinal metabolism (F_g_ = 0.21) and high first pass metabolism (F_h_ = 0.60). High apparent volume of distribution (V/F = 1640 L) was predicted, in accordance to clinical studies. Systemic metabolism mediated by CYP2B6 and CYP3A4 was characterised by a high CL/F of 268 L/h.

**Table 3:** **Simulated artemether pharmacokinetic variables with and without concomitant efavirenz in a cohort of 50 virtual patients compared to observed clinical values**

	Simulated (median and range)		Clinical trial 1 (median and range) (Byakika-Kibwika et al.^2012^)		Clinical trial 2 (geometric mean ± CI) (Huang et al.^2012^)	
	ART	ART + EFV	ART	ART + EFV	ART	ART + EFV
C_max_	30 (11–73)	9 (3–33)	29 (10–247)	12 (2–8)	21.2 (15.2-35)	16.8 (12.0-35.7)
AUC	166 (55–678)	41 (13–228)	119 (26–917)	25 (5–185)	59.5 (40.8-128)	29.4 (23.8-76.6)
CL/F	268 (75–895)	1847 (221–3943)	591 (80–2273)	2558 (414–9960)	-	-
V/F	1604 (100–4719)	4652(1618–8624)	4523 (374–10402)	4715 (1078–28925)	-	-
F_a_	0.86	0.86	-	-	-	-
F_g_	0.21	0.21	-	-	-	-
F_h_	0.60	0.23	-	-	-	-

C_max_, maximum concentrations (ng/ml); AUC: Area Under the Curve (ng.h/ml); CL/F, apparent clearance (L/h); V/F, apparent volume of distribution L/kg; F_a_, fraction of dose absorbed; F_g_, fraction of dose escaping gut metabolism; F_h_, fraction of dose escaping first pass metabolism.

For efavirenz (600 mg once daily), the simulated C_trough_ was equal to 2341 ± 2196 (mean ± SD) vs 1752 ± 1001 ng/ml (reference value), AUC 76282 ± 57204 vs 57592 ± 22849 ng/ml•h, C_max_ 3520 ± 2567 vs 4037 ± 1158 ng/ml (Vrouenraets et al. 2007). Efavirenz mean oral bioavalability, F_oral_, was 0.39, mainly limited by partial dose absorption (F_a_ of 0.45), low intestinal metabolism (F_g_ = 0.95) and low first-pass metabolism (F_h_ = 0.93). Apparent volume of ditribution was 150 L and systemic metabolism, mainly mediated by CYP2B6, CYP1A2, CYP2A6 and CYP3A4, gave a CL/F of 9.7 L/h.

Efavirenz is an inducer of CYP3A4 and CYP2B6 expression with an E_max_ of 6.5 and EC_50_ of 3.9 μM for CYP3A4 and a Ind_max_ of 5.7 and IndC_50_ of 0.8 μM for CYP2B6. In the IVIVE model, efavirenz caused an increase in CYP3A4 and CYP2B6 expression, inducing artemether rate of metabolism with a substantial effect on first pass-metabolism and systemic clearence. Artemether F_h_ was therefore reduced by 60% (from 0.6 to 0.23) and CL/F was increseasd by 6-fold. This had a major effect on artemether pharmacokinetics reducing C_max_ by 60% and AUC by 80%.

A dose increase of artemether was simulated and a dose of 240 mg artemether was predicted to be sufficient to overcome the effect of efavirenz on artemether drug disposition and consequently achieve therapeutic artemether plasma concentrations. At the standard regimen (80 mg twice daily) the simulated AUC of artemether was equal to 166 (55–678) ng.h/mL and the interaction with efavirenz caused a reduction to 41 (13–228) ng.h/mL. A simulated dose increase to 160 mg twice daily gave a median (range) artemether AUC of 77 (25–620) ng.h/ml and a dose increase to 240 mg twice daily resulted in a median (range) artemether AUC of 115 (53–650) ng.h/ml. Artemether is currently coadministered with lumefantrine and EFV has been shown to decrease lumefantrine exposure inducing its metabolism (Byakika-Kibwika et al. 2012).

Factors influencing drug distribution can be investigated using IVIVE, through a full characterisation of the biological processes regulating ADME. Artemether and efavirenz represent two good examples of how a complete knowledge of drug metabolism and physicochemical properties can allow accurate prediction of drug disposition. Efavirenz and artemether metabolism have been investigated *in vitro* and the main enzymes responsible for their clearance have been identified in different studies (Ogburn et al. 2010 ; Takano et al. 2006 ; Ward et al. 2003). Efavirenz is metabolised by several CYPs such as CYP2B6, 1A2, 2A6 and 3A4 and artemether is metabolised in the liver and intestine by CYP3A4 and CYP2B6. Through activation of the nuclear receptor constitutive androstane receptor (CAR), efavirenz strongly influences the expression of several CYPs and transporters. Of particular importance here, CYP2B6 and CYP3A4 activity are induced up to several fold and interactions with CYP3A4 and CYP2B6 substrates have been confirmed in several clinical studies (Esteban et al. 2008 ; Faucette et al. 2007 ; Mouly et al. 2002).

Numerous clinical studies have described poor artemether bioavailability following oral administration but to date a comprehensive measure of its oral bioavailability has not been completed. In our simulation we estimated a F_oral_ equal to 0.11 with a F_a_ of 0.86, F_g_ of 0.21 and F_h_ of 0.6. Slow drug release from tablet and poor solubility are the factors causing an incomplete absorption of the artemether dose. To support these findings, an effect of food on artemether absorption has been described, suggesting how prolonged transit time in the small intestine and increased solubility due to bile acids and fat could increase oral bioavailability (White et al. 1999). CYP isoforms are expressed in the small intestinal tissue and intestinal metabolism has been identified as a major factor defining oral bioavailability of many therapeutic agents, especially CYP3A4 substrates. Artemether bioavailability is also limited by high first-pass metabolism, and the induction of CYP3A4 and CYP2B6 expression by efavirenz in liver tissue is the main cause of decreased artemether F_h_ (0.6 to 0.23) in our simulations. The induction by efavirenz was predicted to have a major impact on the systemic clearance of artemether, increasing plasma CL/F by 6 fold as shown in Table 3. This model suggests that a dose increase to 240 mg of artemether twice daily may correct the effect of efavirenz on artemether pharmacokinetics, restoring sufficient drug exposure. The model presented here provides a rational platform to inform the design for a clinical drug interaction study that may save time and resource while the optimum dose is determined empirically. This study does have some limitations Since artemether is metabolised to DHA, which is also active against *Plasmodium falciparum* with comparable EC_50_ in vitro, then it may be necessary to consider plasma concentrations of DHA to get a complete picture of antimalarial activity (Alin et al. 1990 ; White et al. 1999). Also, efavirenz is a known inducer of some UGT isoforms, such as UGT1A9 and UGT2B7 which also contribute to DHA metabolism (Ilett et al. 2002). Consequently efavirenz is thought to diminish DHA concentrations increasing its rate of metabolism in the liver and kidney. Extensive *in vitro* study aimed to characterise DHA metabolism and distribution may be necessary to develop IVIVE models for the prediction of its pharmacokinetics. Moreover the effect of efavirenz on expression UGTs in the kidney should be investigated to further clarify the effect of EFV on DHA elimination. Efflux and influx transporters can have a big impact upon drug absorption and diffusion into tissues, and might help to explain part of the variability observed in efavirenz and artemether pharmacokinetics. Efavirenz is a strong inducer of *ABCB1* and other transporters. There are currently too many gaps in knowledge of these processes to have incorporated them into the current model. However, *in vitro* investigation of artemether could clarify the role of transporters in this or similar drug-drug interactions (Weiss et al. 2009).

## Conclusion

The developed IVIVE model accurately predicted the pharmacokinetics of efavirenz and artemether and their interaction. The main pharmacokinetic variables for different dosing strategies were simulated and the effect of efavirenz on artemether clearance and bioavailability has been quantified. The clarification of the magnitude of drug-drug interaction between efavirenz and artemether is clinically relevant since sub-therapeutic artemether concentrations can lead to therapeutic failure. The IVIVE approach can be applied to not only to predict the utility of potential dose adjustments but also for the management of drug-drug interactions in special populations such as paediatric, elderly and patients affected by multiple morbidities.

This simulation approach can be viewed as a paradigmatic example demonstrating that IVIVE can be used to investigate clinically relevant ‘what-if’ questions and to inform the design of prospective clinical trials. As previously demonstrated in numerous studies, IVIVE can be used to predict pharmacokinetics in numerous diseases, investigating molecular mechanisms which can impact drug disposition and to inform future clinical studies. Wider application of IVIVE could help researchers gain a better understanding of the molecular mechanisms underpinning variability in drug disposition, an essential condition to further improve the quality of future research projects. Future application of this approach may include other drug-drug interaction simulations, dose optimisation in special populations, prediction of pharmacogenetic effects and optimisation of treatment strategies.

## Abbreviations

ABCB1: ATP-binding cassette B1 transporter
ACT: Artemisinin-based combination therapy
ADME: Absorption, distribution, metabolis and elimination
AIDS: Acquired immune deficiency syndrome
AUC: Area under the curve
BSA: Body surface area
CAR: Constitutive androstane receptor
CAT: Compartmental absorption and transit model
CL/F: Apparent total body clearance of the drug from plasma
CLh: Hepatic clearance
CLint: Intrinsic clearance
Cmax: Maximum contration
CYP3A4: Cytochrome P450 3A4
CYP2B6: Cytochrome P450 2B6
DHA: Dihydroartemisinin
EC50: Concentration causing 50% of maximum induction
EMAX: Maximum induction
Fa: Fraction of dose available for absorption
Fg: Fraction of dose available following intestinal metabolism
Fh: Fraction of dose available following hepatic metabolism
Foral: Oral bioavailability
Fu: Fraction unbound
HAART: Highly active antiretroviral therapy
HIV: Human immunodeficiency virus
Ih: Inducer concentrations in the liver tissue
IVIVE: *in vitro in vivo* extrapolation
NNRTI: Non-nucleoside reverse transcriptase inhibitors
PBPK: Physiologically based pharmacokinetic model
Peff: Effective permeability
Pt:p: Plasma to tissue ratio
QC: Cardiac output
Qh: Hepatic blood flow
Qg: Intestinal blood floow
UGT: Uridine 5'-diphospho-glucuronosyltransferase
V/F: Apparent volume of distribution
